# Localization and Staging of Vascular Adverse Events After Facial Fillers: A Detailed Assessment

**DOI:** 10.1093/asjof/ojaf064

**Published:** 2025-09-23

**Authors:** Sara Khoshnaw, Leonie Schelke, Gillian Murray, Peter J Velthuis

## Abstract

**Background:**

Vascular adverse events (VAEs) are among the most feared complications following filler injections. At the specialized filler-complication clinic, the authors observed that the lateral region of the face experiences a lower risk of VAE-associated necrosis compared with the medial region.

**Objectives:**

The authors of this study aim to determine whether the medial facial region has a higher risk of necrosis following dermal filler injections compared with the lateral facial region. To assess this observation statistically, the authors analyzed all VAE cases reported between 2019 and 2024.

**Methods:**

A retrospective analysis was undertaken. High-resolution photographs enabled precise anatomical localization and accurate staging of the VAEs. The face was anatomically subdivided utilizing 3 separate classification methods: (1) facial zones based on the 4 primary arteries (ophthalmic, superficial temporal, maxillary, and facial arteries); (2) classification based on whether subzones were supplied by branches of the external carotid artery or by both the external and internal carotid arteries; and (3) categorization into the medial or lateral region of the face, based on their relative location to the line of ligaments. The clinical patterns of VAEs were classified into 5 stages, distinguishing between non-necrotic (Stages 1 and 2) and necrotic (Stages 3-5) outcomes, reflecting a stepwise clinical development of symptoms over time. Statistical analyses, including *χ*^2^ and Fisher's exact tests, were utilized to evaluate the distribution of VAE stages within each of the 3 anatomical classification methods.

**Results:**

In total, 120 patients with documented VAEs between 2019 and 2024 were included. Necrotic VAEs (Stages 3-5) occurred significantly more frequently in the medial facial region (*P* = .048). No significant correlation was found between these stages and distributions of both carotid artery branches nor the primary facial arteries.

**Conclusions:**

In this study, the authors highlight a higher risk of necrosis following dermal filler treatments in the medial facial region compared with the lateral region. A correlation between necrosis and the distribution of facial primary arteries or the carotid arteries seems to be absent, suggesting that local factors (eg, number or function of anastomoses/choke anastomoses) may play an important role.

**Level of Evidence: 4 (Therapeutic):**

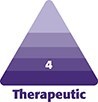

Since the 1990s, several resorbable fillers have been developed and introduced, including different brands of hyaluronic acid (HA) and biostimulating fillers, such as calcium hydroxyl-apatite and poly-L-lactic acid. These fillers came after earlier bovine collagen and avian HA formulations.^1,2^ The first reported case of vascular adverse events (VAEs) related to fillers was documented in 2002.^3^ Between 2000 and 2020, the number of published photographs of filler-induced skin ischemia and necrosis increased 30-fold, leading to an increased interest in understanding the risk factors and clinical presentations of VAEs following dermal filler treatments.^4^

Possible related risk factors for VAEs include the use of needles instead of cannulas.^5,6^ However, although blunt cannulas are generally considered safer, they may still perforate arterial walls under certain conditions.^7,8^ Patients with a history of nasal surgery undergoing nasal filler treatments are also at an increased risk of VAEs.^9^ Additionally, the potential for fillers to expand in volume by 100% to 700% increases their occlusive potential and, consequently, the risk of VAEs.^10,11^ Lastly, injection pressure during filler procedures plays a crucial role, as lowering the injection pressure can help minimize the risk of VAEs during treatment.^12^

A 2021 FDA panel reported 411 unique cases of serious vascular complications related to filler use between August 1, 2015, and August 1, 2020. Of these, 79% occurred following injections in the medial facial region, including zones such as the perioral/lips, nasolabial folds, nose, glabella, chin, forehead, and marionette lines.^13^ Literature reviews evaluating VAEs in the face predominantly highlight the medial facial region, including the glabella, nose, nasolabial folds, lips, and chin, as being high risk for VAEs.^14^ In 2011, the dermatology department at our hospital opened a specialized outpatient clinic to manage filler-related complications. In 2019, the first 21 cases with VAEs were published.^15^ In the following years until 2024, >120 patients have been referred for management. Clinical observations indicate that severe cases of VAEs are more frequently observed in the medial facial region compared with the lateral region. By systematically analyzing the occurrence of the different clinical stages of VAEs, particularly the presence of necrosis across different facial zones, this study aims to investigate whether the association between localization, as an expression of the artery involved, and more severe stages of VAEs after facial filler treatments is statistically significant.

## METHODS

### Patient Selection and Data Collection

A retrospective analysis of documented VAEs was undertaken. Patients were identified through a detailed review of medical records from a specialized complication clinic in the Netherlands from October 2019 to March 2024. Patients were included if they presented with VAEs in the face after dermal filler treatments. The diagnosis of VAEs was based on the clinical picture, which included a combination of symptom assessment, duplex ultrasound examination, high-resolution photographic evidence, and information from the treating physician and referral letter, all of which were documented in the patient records.

High-resolution photographs from patient records were used to assess the anatomical distribution and staging of the VAEs. To ensure consistent evaluation, 2 independent physicians (S.K. and L.S.S.) reviewed all photographs for anatomical distribution and severity staging, with a third physician (P.J.V.) giving a decisive vote in cases of disagreement. Data collected included patient demographics, anatomical location of VAEs, and clinical characteristics of each adverse event. All patient data were anonymized to protect confidentiality.

To ensure dataset homogeneity and emphasize clinically relevant vascular complications, VAEs affecting the oral cavity were excluded from our study. Unlike facial skin, the oral cavity often lacks the ability to form blisters or pustules because of the thin epithelium of the mucosa. This thin epithelium causes superficial tissue damage to progress rapidly into erosions, bypassing the blister or pustule stage commonly seen on the skin. The tongue lacks hair and sebaceous glands, which contributes to a distinct tissue response in VAEs. VAEs in the temple region presenting solely as hair loss were excluded as well, because alopecia is not a feature observed in other zones of the face.

Ethical compliance was ensured through the informed consent process, securing voluntary participation, and providing clear information about the study's objectives. The research was approved by the ethical committee of Erasmus MC (MEC-2020-0150).

### Anatomical Stratification and Subtyping

The face was anatomically subdivided using 3 separate classification methods.

First, 4 facial zones were defined based on the primary branches supplying blood flow from both the external and internal carotid arteries. This included the ophthalmic, superficial temporal, maxillary, and facial artery branches. Each zone was further subdivided into subzones, reflecting the blood supply by the branches of the 4 arteries to include the subzone typically affected (Table 1).

**Table 1.** Division of the Face into Zones and Associated Subzones, Based on the Primary Branches of the External and Internal Carotid Arteries

Zone	Subzones
Ophthalmic	Supraorbital, supratrochlear, dorsal nasal, zygomaticofacial
Superficial temporal	Superficial temporal, transverse facial
Maxillary	Infraorbital
Facial	Facial, lateral nasal, superior labial, inferior labial, mental

**Figure 1.**
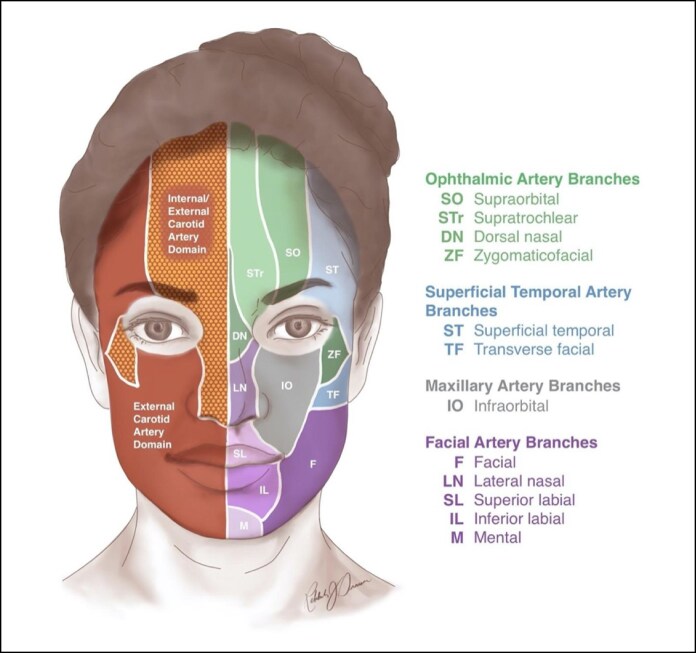
Anatomical illustration used in this study for the 3 separate classification methods.

Second, the subzones defined in the first analysis were reclassified based on their arterial supply into 2 categories: those supplied by branches of the external carotid artery, and those receiving blood from both external and internal carotid arteries (Figure 1). This classification acknowledges the overlapping vascularization in some subzones.

Third, the subzones were classified as medial or lateral region of the face based on their location relative to the line of facial ligaments, with the zygomatic ligament used as the most caudal reference point. The lateral region included the superficial temporal, transverse facial, and zygomaticofacial subzones, whereas the remaining subzones were classified as the medial region of the face.

### Classification of Vascular Event Severity

To standardize the assessment of vascular event over time following dermal injectable treatments, the staging system of Murray et al (Guideline for the Management of Hyaluronic Acid Filler–induced Vascular Occlusion) was applied.^16^ This 5-stage system is designed to illustrate the skin signs over time. Stage 1 is marked by interrupted blood flow, which manifests as pallor because of a transient reduction in perfusion. In Stage 2, a livedoid erythema pattern appears. This may result from direct trauma to the vessel or reactive vascular spasm, both leading to perfusion deficits in the associated angiosome. Because the condition progresses to Stage 3, necrotic changes such as blisters and pustules emerge, driven by the accumulation of leukocytes at the site of injury. Stage 4 is characterized by wounds and erosions as dermal and epidermal tissue sloughs away because of prolonged oxygen deprivation. By Stage 5, the tissue becomes fully devitalized, resulting in the formation of black eschar (Table 2).

**Table 2.** The 5-Stage Classification System of Vascular Adverse Events Over Time

Stage	Clinical signs	Pathophysiology
1	Non-necrotic: pallor	Interrupted blood flow
2	Non-necrotic: livedoid erythema pattern	Blood vessel laceration, vascular spasm in angiosome
3	Necrotic: pustules and/or blisters	Leukocytes accumulation, sterile +/− including bacterial overgrowth
4	Necrotic: wounds and/or erosions	Remnants of blisters or dermal/epidermal sloughing resulting from anoxia
5	Necrotic: black eschar	Devitalized tissue

Adapted from Murray et al.^16^

### Statistical Analysis

Descriptive statistics, such as absolute frequencies and relative percentages, were used to illustrate the spread of VAEs across the different facial zones.

All data were processed using the Statistical Package for the Social Sciences 29.0 (SPSS; IBM, Armonk, NY). Statistical analysis focused on determining associations between the anatomical localization of VAEs and the occurrence of necrosis. Chi-square and Fisher's exact tests were applied to assess differences in the incidence of necrosis between the medial and lateral regions of the face, as well as the relationship between necrosis and the arterial branches involved. A 1-sided *P*-value of ≤.05 was considered statistically significant.

## RESULTS

### Demographics and Patient Characteristics

This retrospective study evaluated a cohort of 120 patients (98 females, 22 males) with a mean age of 40 years (range, 18-77 years).

### Staging of Vascular Adverse Events (VAEs)

**Figure 2.**
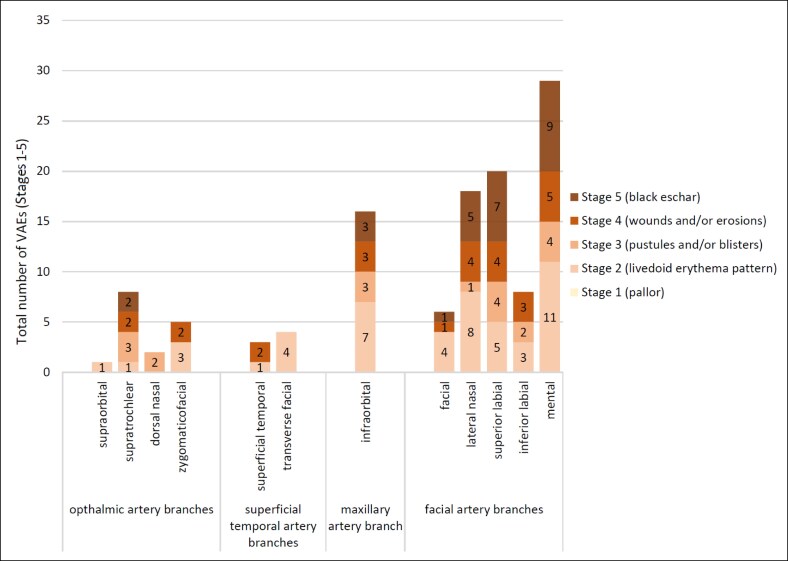
Absolute number of vascular adverse event stages in postfiller complications for the 4 major facial arteries and their branches.

The VAE classification provided insight into the severity of VAEs. A total of 59.2% (*n* = 71) of cases exhibited necrotic outcomes (Stages 3-5). Stages 4 and 5 accounted for the highest number of necrotic cases, both 36.6% (*n* = 26), followed by Stage 3 at 26.8% (*n* = 19). Figure 2 details the precise location of each VAE within the subzone and the corresponding staging, offering a comprehensive overview of the distribution and severity of VAEs in the facial landscape.

### Anatomical Distribution of VAEs

The anatomical distribution of VAEs was analyzed using 3 separate classification methods.

First, based on the 4 primary arterial zones—defined by the primary branches of the external and internal carotid arteries (facial, ophthalmic, maxillary, and superficial temporal)—the majority of VAEs (68%, *n* = 81) occurred in the facial artery zone, followed by 13% (*n* = 16) each in the ophthalmic and maxillary zones, and 6% (*n* = 7) in the superficial temporal zone. Among these, the proportion of necrotic VAEs (Stages 3-5) was highest in the ophthalmic zone (69%, *n* = 11) and facial artery zone (62%, *n* = 50), followed by the maxillary zone (56%, *n* = 9) and superficial temporal zone (29%, *n* = 2).

Second, when classifying subzones based on their vascular supply, 72% of VAEs (*n* = 86) occurred in areas supplied solely by the external carotid artery, whereas 28% (*n* = 34) occurred in areas with dual supply from both the external and internal carotid arteries. Necrosis was present in 59% (*n* = 51) of the external carotid artery group and in 62% (*n* = 21) of the dual-supply group.

Third, according to the medial vs lateral facial region classification—based on anatomical boundaries defined by the line of facial ligaments—VAEs occurred predominantly in the medial region (90%, *n* = 108), with only 10% (*n* = 12) located laterally. Notably, necrosis was observed in 63% (*n* = 68) of medial cases compared with 33% (*n* = 4) in the lateral region.

**Figure 3.**
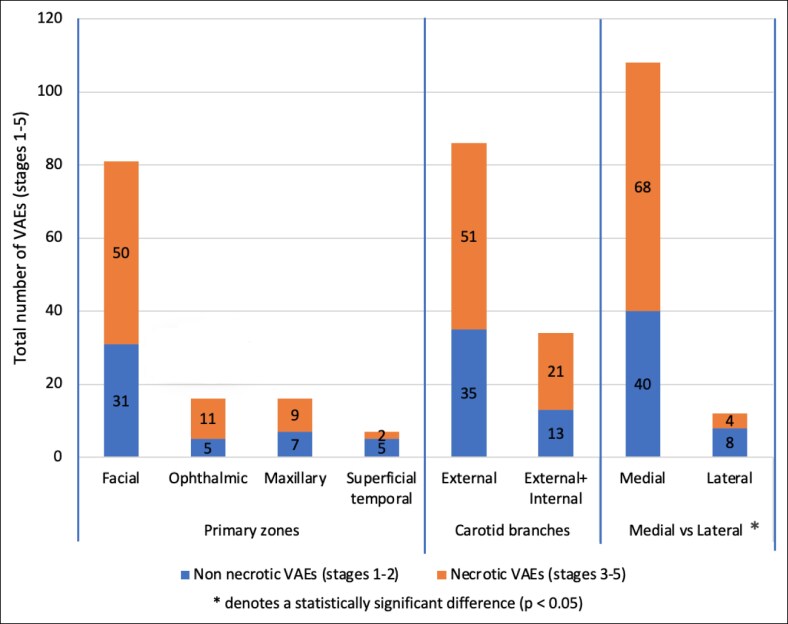
Absolute numbers of vascular adverse event stages across the 3 anatomical classification methods.

The total number of VAEs and necrotic outcomes across these 3 anatomical classification systems are visualized in Figure 3.

### Statistical Analysis

There was no significant correlation in the frequency of occurrence of necrosis and the 4 primary zones (*P* = .31).

Nor was there a significant correlation between the frequency of occurrence of necrosis and the arterial branches of either the internal or external carotid arteries (*P* = .49).

Utilizing the Fisher's exact test, a significant difference in the frequency of occurrence of necrosis between the medial and lateral regions of the face was calculated (*P* = .048).

## DISCUSSION

This study found a higher risk for more severe filler-related VAEs in the medial region of the face when compared with the lateral. There were no significant associations with the 4 primary facial arteries, or branches of the internal or external carotid arteries. This suggests that factors beyond primary arterial supply may contribute to the occurrence and severity of VAEs. It is of interest to speculate on the pathophysiology of this phenomenon. There may be an association with choke anastomoses, because their number, course, or functionality may differ between facial subzones.

The literature describes filler-induced vascular occlusion as a multifactorial cascade, involving vaso-canulation, vaso-inoculation, vaso-dissemination, and vaso-occlusion, all leading to reduced tissue perfusion and ischemia.^15^ Other studies suggest that vascular spasms of choke anastomoses may also contribute to the clinical presentation of filler-related VAEs.^17,18^ Anastomoses are permanent vascular connections between 2 angiosomes, perforasomes, or arteriovenous shunts, ensuring consistent blood flow regardless of changes in perfusion pressure.^19^ In the event of vascular obstruction during dermal filler injections, these anastomoses can dilate to maintain adequate blood flow.^20^ Choke anastomoses are small, vascular connections between adjacent vascular regions. At rest, they have a limited diameter and minimal blood flow, acting as an emergency reserve that becomes active only when the primary blood supply is compromised.^21^ Choke anastomoses can dilate but can also reduce the severity of complications by reflexively limiting blood flow to adjacent vascular regions through constriction, thereby minimizing the extent of tissue affected.^20^

HA fillers present within a vessel can act as a noxious stimulus, causing an irritant response that leads to the collapse of choke anastomoses and vascular compromise in adjacent angiosomal regions.^22-24^ Choke anastomoses also act as a shunt converter, which results in necrosis of the superficial dermis while preserving survival of the deep cutaneous adipose tissue.^25^ The functioning of choke anastomoses may contribute to decreased tissue perfusion in cases of VAEs.^26,27^ A hypothesis for the higher risk of VAEs in the medial facial region is the greater prevalence of choke anastomoses in the medial facial region compared with the lateral region. Another hypothesis is that the lateral region of the face benefits from a greater number of anastomotic connections between different arterial branches, which could offer improved collateral circulation when a VAE occurs. However, further research is needed to confirm this.

The clinical images associated with filler-induced VAEs have been incorporated into the 5-point staging system described by Murray et al.^16^ This system accounts for the temporal progression of VAEs. The initial stages—blanching and reticulated skin patterns—typically manifest within the first 2 days. The later stages, characterized by clear signs of skin necrosis, generally appear by Days 3 or 4 or even later. Stages 3 and 4 may occur in reversed order. In our study, we did not observe cases of tissue blackening because of coagulative necrosis as mentioned in the study by Murray et al. Instead, we identified 26 cases involving erosions and wounds, which we categorized as Stage 4 under the staging system. Stage 3, defined as pustulosis, is attributed to bacterial overgrowth but does not inherently indicate bacterial infection. Histological and microbiological analyses have shown that 50% of filler-induced skin necrosis cases revealed no bacterial growth in cultures.^28^ In a separate study, histological examination of a biopsy from a necrotic case tested negative for periodic acid–Schiff staining, commonly used to detect fungi, polysaccharides, or specific bacteria.^29^ These findings suggest that many necrotic cases may result from sterile processes, such as ischemia-induced tissue death, rather than infectious causes. Notably, conditions such as psoriasis, pyoderma gangrenosum, and certain forms of acne can also produce pustules and blisters without bacterial involvement.^30,31^ We therefore interpret Stage 3 as an early stage of necrosis, because minimal pitted atrophic lesions were eventually observed in all these patients.

When a patient presents with a filler-induced VAE, treatment is typically initiated immediately to prevent progression to later stages. However, a recent case report described a patient with clinical signs of VAE following HA filler injection who did not receive any specific treatment, yet the condition resolved spontaneously.^32^ Based on the severity, extent, and progression of symptoms over time, the CMAC Reference Group has recommended conservative management for a small subset of patients, particularly those in Stages 1 and 2 with mild involvement and no progression over several days. These cases have shown no progression to necrosis (Stages 3-5). Fundamental studies further support that ischemic skin changes, such as pallor and reticulation, do not necessarily lead to necrosis.^33^ Taken together, this evidence suggests that clinical signs of VAE do not invariably progress to necrosis.

The retrospective nature of this study and reliance on data from a single specialized medical center limit the strength of the evidence presented. Additionally, recognition bias or sampling errors may have influenced the observed patterns of VAEs. Because of its retrospective setup, the time period after injection could not be taken into account. It is important to note that the photographs reviewed in this study were taken at the time of presentation to our clinic. In all cases, patients had already been seen by their original treating physician, and treatment may have been attempted before referral. As a result, there was typically a delay between symptom onset and clinical assessment at our center. This delay may have influenced the clinical staging, because early features such as pallor (Stage 1) were no longer visible by the time of documentation. Additionally, because of referrals from various external practitioners, demographic and clinical background data (eg, comorbidities, smoking history, and previous surgery) were often incomplete and could not be included in the analysis, limiting risk profile assessment.

Furthermore, not all patients with VAEs in the Netherlands may have been referred to our clinic. This could have led to underreporting of milder cases, creating a selection bias toward more severe presentations. Another limitation is that the frequency of filler treatments per facial region was not recorded. Our study reports absolute numbers of VAEs per anatomical subzone but lacks data on the total number of injections performed in each subzone, as well as on the exact injection site that led to the VAE. Without this denominator, it is not possible to determine the relative risk of VAEs per injection site.

To minimize the risk of VAEs during filler procedures, the use of Doppler ultrasound for both arterial mapping and guiding filler injections is recommended.^34,35^ Ultrasound can help identify abnormal vascular structures before the injection, thus reducing the risk of vascular injury.^36^ The diagnosis of vascular occlusion following filler treatment is primarily clinical but may be supported by Doppler ultrasound imaging too.^37^ As described by Schelke et al, Doppler ultrasound can reveal areas of increased vascularity—interpreted as collateral arteries—adjacent to a “silent area,” which shows little to no detectable vascular structures.^38^ HA filler typically does not appear as an anechoic or hypoechoic pocket but rather as an ill-defined hypo- to isoechoic spot within the silent area.

## CONCLUSIONS

In this retrospective study, the authors demonstrate a significantly higher occurrence of severe VAEs (Stages 3-5) following dermal filler treatment in the medial facial region compared with the lateral region. No significant associations were found between the occurrence of necrosis and the anatomical distribution of the primary facial arteries or branches of the internal and external carotid arteries.
